# SMARCAD1 is an ATP-dependent stimulator of nucleosomal H2A acetylation via CBP, resulting in transcriptional regulation

**DOI:** 10.1038/srep20179

**Published:** 2016-02-18

**Authors:** Masamichi Doiguchi, Takeya Nakagawa, Yuko Imamura, Mitsuhiro Yoneda, Miki Higashi, Kazuishi Kubota, Satoshi Yamashita, Hiroshi Asahara, Midori Iida, Satoshi Fujii, Tsuyoshi Ikura, Ziying Liu, Tulip Nandu, W. Lee Kraus, Hitoshi Ueda, Takashi Ito

**Affiliations:** 1 Nagasaki University School of Medicine, Nagasaki 852-8523, Japan; 2 Daiichi Sankyo RD Novare CO., LTD., Tokyo 134-8630, Japan; 3 Tokyo Medical and Dental University, Tokyo 113-8510, Japan; 4 Kyushu Institute of Technology, Fukuoka 820-8502, Japan; 5 Radiation Biology Center, Kyoto University, Kyoto 606-8501, Japan; 6 UT Southwestern Medical Center, Dallas, TX, 75390, USA; 7 Okayama University, Okayama 700-8530, Japan

## Abstract

Histone acetylation plays a pivotal role in transcriptional regulation, and ATP-dependent nucleosome remodeling activity is required for optimal transcription from chromatin. While these two activities have been well characterized, how they are coordinated remains to be determined. We discovered ATP-dependent histone H2A acetylation activity in *Drosophila* nuclear extracts. This activity was column purified and demonstrated to be composed of the enzymatic activities of CREB-binding protein (CBP) and SMARCAD1, which belongs to the *Etl1* subfamily of the Snf2 family of helicase-related proteins. SMARCAD1 enhanced acetylation by CBP of H2A K5 and K8 in nucleosomes in an ATP-dependent fashion. Expression array analysis of S2 cells having ectopically expressed SMARCAD1 revealed up-regulated genes. Using native genome templates of these up-regulated genes, we found that SMARCAD1 activates their transcription *in vitro*. Knockdown analysis of SMARCAD1 and CBP indicated overlapping gene control, and ChIP-seq analysis of these commonly controlled genes showed that CBP is recruited to the promoter prior to SMARCAD1. Moreover, *Drosophila* genetic experiments demonstrated interaction between SMARCAD1/*Etl1* and CBP/*nej* during development. The interplay between the remodeling activity of SMARCAD1 and histone acetylation by CBP sheds light on the function of chromatin and the genome-integrity network.

The eukaryotic genome is packaged into the higher-order DNA–protein structure chromatin. The nucleosome, the basic unit of chromatin, is composed of an octamer of core histones (two each of H2A, H2B, H3, and H4), around which 146 base pairs of DNA are wrapped^1^,^2^. Nucleosomes act as general repressors of transcription, and transcription from the repressed templates is activated by the actions of transcriptional activators, histone modifiers, and chromatin remodeling complexes^3^,^4^,^5^,^6^.

Transcriptional regulation from chromatin is a dynamic process, accompanied by histone modifications such as methylation, phosphorylation, ubiquitylation, and acetylation^7^,^8^,^9^,^10^,^11^,^12^,^13^. Among these posttranslational modifications, histone acetylation plays a pivotal role in transcriptional regulation. Historically, histone acetyl transferases (HATs) have been grouped into nuclear A-type HATs or cytoplasmic B-type HATs. Although B-type HATs, such as Hat1, are related to newly synthesized core histones, A-type HATs are related to nuclear events, such as transcription and DNA repair^14^. Due to their similarity in several homologous regions and acetylation-related motifs, HATs are further subdivided into several families, such as the Gcn5-related N-acetyltransferases (GNATs; Gcn5 and PCAF), the MYST acetyltransferases (MOZ, Ybf2/Sas3, Sas2, and Tip60), the p300/CBP transcriptional coactivators (p300 and CBP), the general transcription factor TAF1 (also known as TAFII250), and nuclear receptor coactivators (SRC-1, ACTR, and TIF2)^12^. Among these diverse HATs, the family members p300 and CBP are central regulators of transcription, with roles as global coactivators in higher eukaryotes. Tight regulation of p300 is critical for ensuring precise histone acetylation and gene activation^15^,^16^.

By contrast, using a different class of chromatin modifiers, chromatin remodeling activity utilizes the energy of ATP to move nucleosomes along the DNA strand and remove or release them from their interaction with DNA. Thus, ATP-dependent nucleosome remodeling activity is required for optimal transcription from chromatin^4^,^5^,^6^,^17^,^18^,^19^,^20^. The catalytic subunits of ATP-dependent chromatin remodeling complexes belong to the Snf2 family of helicase-related proteins found in all eukaryotes. Snf2 family members are divided into 24 distinct subfamilies, including Swr1, EP400, INO80 and Etl1, which belong to the swr1-like grouping, based on sequence alignment of the helicase-related regions^21^.

Histone modifications, such as acetylation, that are closely related to transcription should function together with ATP-dependent chromatin remodeling activity, since conformational changes of chromatin are essential for transcription. However, compared with the extent of knowledge about these two activities considered separately, there is little evidence concerning their coordination in transcriptional regulation. It is known that the human Mi-2-NuRD complex couples chromatin remodeling ATPase activities with histone deacetylation enzymatic functions^22^. By contrast, in this study we found ATP-dependent nucleosomal core histone acetylation activity in *Drosophila* nuclear extracts. We purified this activity and determined that it is composed of p300/CBP transcriptional coactivators and SMARCAD1, a member of the Snf2 family of helicase-related proteins. We established that SMARCAD1 functions with CBP and regulates transcription *in vivo* and *in vitro*. Our results underscore the interplay between remodeling activities and histone modifiers in the function of chromatin and the genome-integrity network.

## Results

### Discovery of ATP-dependent H2A acetylation activity and identification of CBP and SMARCAD1

**Figure 1:**
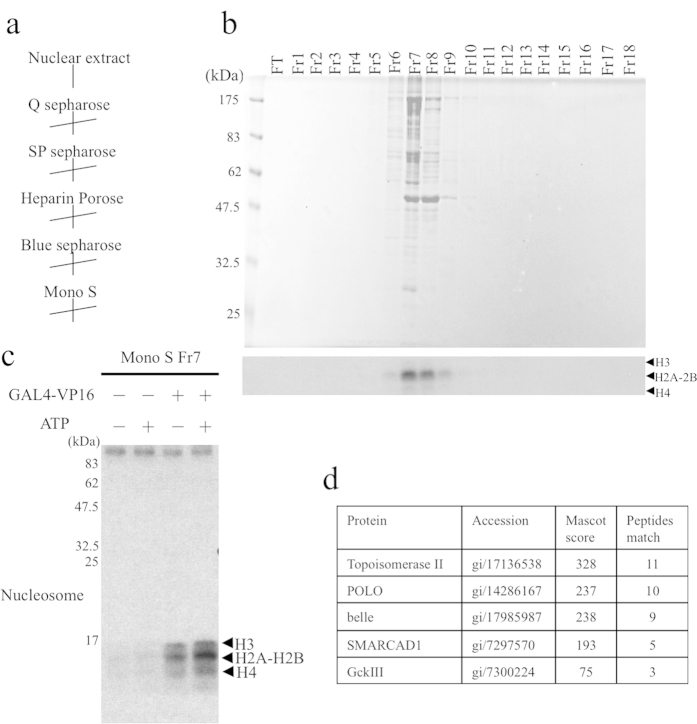
SMARCAD1 is an ATP-dependent stimulator of the nucleosomal acetyltransferase CBP. (**a**) Fractionation scheme of ATP-dependent histone H2A acetylation activity using chromatin reconstituted with purified *Drosophila* core histones and pGIE0 plasmid DNA, which has GAL4 binding sites upstream of the AdE4 promoter. (**b**) CBB staining of an SDS-PAGE gel (Top) and histone acetyltransferase (HAT) assay of the fractions derived from the final Mono S column chromatography of the purified fractions (Bottom). The HAT assay was performed in the presence of [^3^H]-acetyl-CoA. (**c**) Active fractions were incubated with chromatin, GAL4-VP16, and ATP in the presence of [^3^H]-acetyl-CoA, as indicated. (**d**) Candidate ATP-utilizing proteins whose fragment sequences were identified by LC–MS/MS from the top to the bottom of the gel.

Using a crude *Drosophila* nuclear extract, we detected ATP-dependent acetylation of histone H2A. After reconstituting chromatin using salt dialysis, we used a pGIE0 plasmid having five GAL4 binding sites upstream of the adenovirus E4 promoter as a DNA template for the nucleosomal histone acetylase assay^23^,^24^,^25^. For this assay, we purified ATP-dependent nucleosomal histone acetylase activity using column chromatography, as indicated in Fig. 1a. Final purification of this activity using a Mono S column is shown in Fig. 1b. Fraction 7 acetylates mainly the core histone H2A in the presence of the GAL4-VP16 transcriptional activator and ATP (Fig. 1c). Next, we loaded Fraction 7 onto an SDS-PAGE gel and analyzed the gel from top to bottom by liquid chromatography–tandem mass spectrometry (LC–MS/MS), since it was not pure enough to identify the corresponding band. We obtained two trypsin-generated peptide sequences (TALLPTLEK and LGFDIDDGSALADHK) that were identical to segments of *Drosophila* CBP/*nej* (FBgn0261617). CBP/*nej* is the only protein that is categorized as a histone acetylase among the sequences obtained. Since the acetylation activity is ATP dependent, we focused on ATP-utilizing molecules, such as kinases and helicases, in addition to acetylases. We also obtained several sequences that are identical to topoisomerase II, polo kinase, belle kinase, SMARCAD1, and GckIII (Fig. 1d, Supplementary Table 1). We expressed and purified all of these enzymes in Sf9 cells and tested their activity. We found that only SMARCAD1 has activity facilitating histone H2A acetylation by CBP/p300. Thus, we concluded that SMARCAD1 is an ATP-dependent stimulator of the nucleosomal acetyltransferase CBP.

### Recombinant SMARCAD1 stimulates histone H2A acetylation by recombinant human CBP/p300

**Figure 2:**
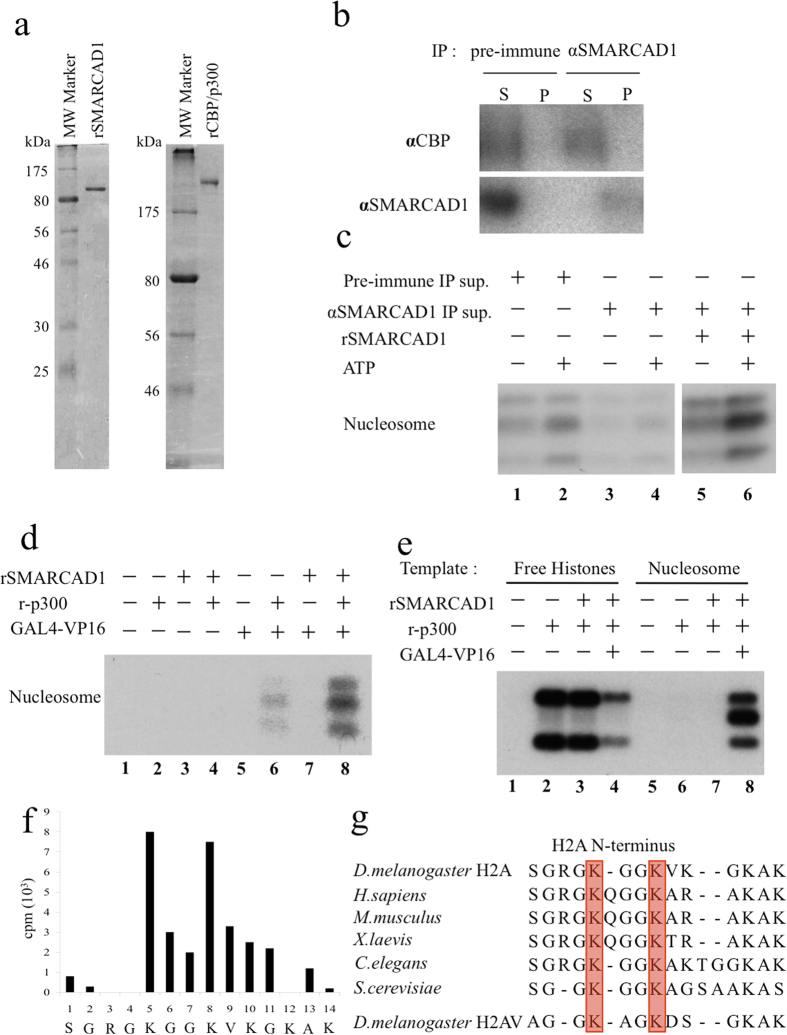
SMARCAD1 and p300/CBP function together to acetylate H2A *in vitro*. (**a**) SDS-PAGE gel analysis of affinity-tag-purified recombinant SMARCAD1 and p300 expressed in Sf9 cells. (**b**) Immunoprecipitation (IP) studies of SMARCAD1 with an SP column fraction after Q sepharose purification of a nuclear extract from Drosophila embryos as shown in Fig. 1a. Supernatants (S) or pellets (P) of IP with pre-immune antibodies or anti-SMARCAD1 antibodies were resolved on SDS-PAGE gels and subjected to immunoblotting with anti-SMARCAD1 or anti-CBP antibodies, as indicated. (**c**) A HAT assay was done using the supernatant of IP with pre-immune sera (pre-immune IP sup.) or anti-SMARCAD1 antibodies (anti-SMARCAD1 IP sup.) corresponding to Fig. 2a in the presence or absence of ATP and recombinant SMARCAD1, as indicated. (**d**) HAT assay of a chromatin template in the presence or absence of SMARCAD1, p300, and GAL4-VP16, as indicated. (**e**) HAT assays using purified core histones or chromatin as substrates in the presence or absence of SMARCAD1, p300, and GAL4-VP16, as indicated. (**f**) Acetylating core histones using the active fraction from a POROS heparin column, and the acetylated histones were separated using SDS-PAGE and blotted onto a PVDF membrane. The band corresponding to H2A and H2B was then analyzed by Edman degradation and liquid scintillation counting. (**g**) Sequence alignment of histone H2A N-terminal tails from yeast to human. Two red boxes indicate evolutionarily conserved lysines 5 and 8 (or 9).

SMARCAD1 was expressed in Sf9 cells and purified with Flag-tag purification. Human p300 was expressed in SF9 cells and affinity-purified with Ni-NTA resin as previously described (Fig. 2a)^24^,^26^. The specificities of anti-SMARCAD1 and anti-CBP antibodies were confirmed by western blotting using whole *Drosophila* cell lysates, and bands detected by these antibodies were reduced by SMARCAD1 knockdown or CBP knockdown (Supplementary Figs 1a and 3b). We immunoprecipitated SMARCAD1 from an SP column fraction after Q sepharose column purification of a nuclear extract (Fig. 2b). After immunoprecipitation with anti-SMARCAD1 antibodies, CBP remained in the supernatant, while SMARCAD1 was precipitated as shown in Fig. 2b Thus, CBP does not make a tight complex with SMARCAD1. After immunodepletion, the ATP- dependent stimulation of histone acetylation activity is lost in the anti-SMARCAD1 immunoprecipitation supernatant (Fig. 2c, lanes 3, 4). Recombinant SMARCAD1 (rSMARCAD1) stimulates acetylation of the nucleosomal histone H2A by native CBP in an ATP-dependent manner, an activity that is retained in the supernatant after anti-SMARCAD1 immunoprecipitation (Fig. 2c lanes 5, 6). rSMARCAD1 also stimulates acetylation of the nucleosomal histone H2A by recombinant p300 (r-p300) in an ATP-dependent manner (Fig. 2d). r-p300 acetylates free histone, irrespective of the presence or absence of rSMARCAD1 (Fig. 2e lanes 2, 3). Furthermore, r-p300 acetylates mainly histones H3 and H4 when free histones were used as a substrate (Fig. 2e lanes 2–4). Interestingly, free histone H2A/H2B was not strongly acetylated by r-p300, as GAL4-VP16 somewhat inhibits this reaction in the absence of a DNA template (Fig. 2e lane 4). Chromatin inhibits core histone acetylation by r-p300, and overcoming this inhibition, GAL4-VP16 and rSMARCAD1 mainly stimulate acetylation of H2A/H2B by r-p300 (Fig. 2d lanes 6, 7 and Fig. 2e). To confirm the specific acetylation sites within the core histone tails that are acetylated by CBP in cooperation with SMARCAD1, we performed a HAT assay using chromatin assembled with DNA and native core histones or recombinant core histones as a substrate, and acetylated histones were subjected to Edman degradation followed by sequence analysis in parallel with liquid scintillation counting. The [^3^H]-acetyl group is mainly incorporated into H2A/H2B rather than H3 and H4, so we focused on acetylation of H2A/H2B. Since H2A and H2B migrate closely on SDS-PAGE, we sequenced both of them together. It is known that the N-terminus of native H2A is blocked by acetylation^27^,^28^. However, when H2A is expressed in bacteria, we confirmed that the first residue (methionine) is cleaved almost completely, resulting in expression starting from the second residue, serine, as shown in Supplementary Fig. 2a. Since we could determine the sequence of bacterially expressed H2A by Edman analysis, the N-terminus of H2A is not blocked by acetylation. On the other hand, it is known that the N-terminus of native H2B is not blocked. In addition, the N-terminus of bacterially expressed H2B is partially cleaved, resulting in translation starting from both the first residue (methionine) and the second residue (proline) (Supplementary Fig. 2b). [^3^H]-acetate was not released from native H2A/H2B (Supplementary Fig. 1e; the average number of counts for the first 10 residues is 1.63 × 10^3 ^cpm, and there are no significant peaks, suggesting that there has been no cleavage of acetylated residues). However, [^3^H]-acetate was released from acetylated bacterial H2A/H2B (Fig. 2f, 8.0 × 10^3 ^cpm for the 5th residue and 7.5 × 10^3 ^cpm for the 8th residue). These results indicate that [^3^H]-acetate is incorporated into H2A. Because ^3^H was released at K5 and K8, we concluded that CBP and SMARCAD1 mainly stimulate histone H2A K5 and K8 (Fig. 2f). These two residues are well conserved among species (Fig. 2g).

### In early-stage embryos, SMARCAD1 is constitutively expressed and localized to the nucleus

**Figure 3:**
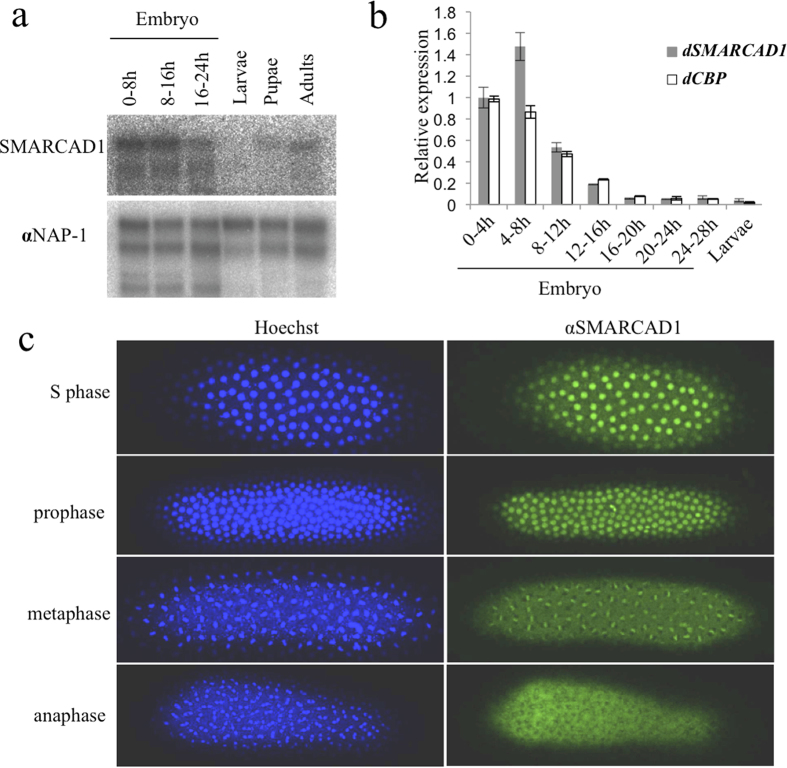
SMARCAD1 is localized in the nucleus during early embryogenesis. (**a**) SMARCAD1 protein levels were analyzed by western blotting with anti-SMARCAD1 antibodies using whole-cell extracts from different developmental stages. The signals obtained with anti-NAP-1 antibodies were used as loading control. (**b**) The expression profiles of SMARCAD1 and CBP at different times of embryonic development were monitored by RT-qPCR. The expression of SMARCAD1 and CBP were normalized to that of *dAct79B* at each developmental stage. (**c**) Immunostaining with anti-SMARCAD1 antibodies (green) during the cell cycle of the early embryonic stage (0–1 h). DNA was stained with Hoechst 33342 dye (blue).

Analysis of western blotting and immunostaining of SMARCAD1 indicated that it is constitutively expressed in early-stage embryos (Fig. 3a) and is located in the nucleus, suggesting a role there (Fig. 3c). During early developmental stages, the expression patterns of CBP and SMARCAD1 look similar (Fig. 3b). SMARCAD1 is also expressed in adult cells as well as in early-stage embryos, suggesting that it contributes not only to embryogenesis but also to biological phenomena such as transcription in the adult (Fig. 3a). Based on its protein sequence and domain, SMARCAD1 is classified in the *Etl1* subfamily of the Snf2 family. The Snf2 family contains a helicase motif that separates nucleic acid strands and translocates DNA in an ATP-dependent fashion^6^,^21^. Multiple sequence alignment of the helicase-related region indicates that 24 distinct subfamilies exist^21^, including Snf2, Iswi, and *Etl1*.

### SMARCAD1 activates transcription *in vivo* and *in vitro*

**Figure 4:**
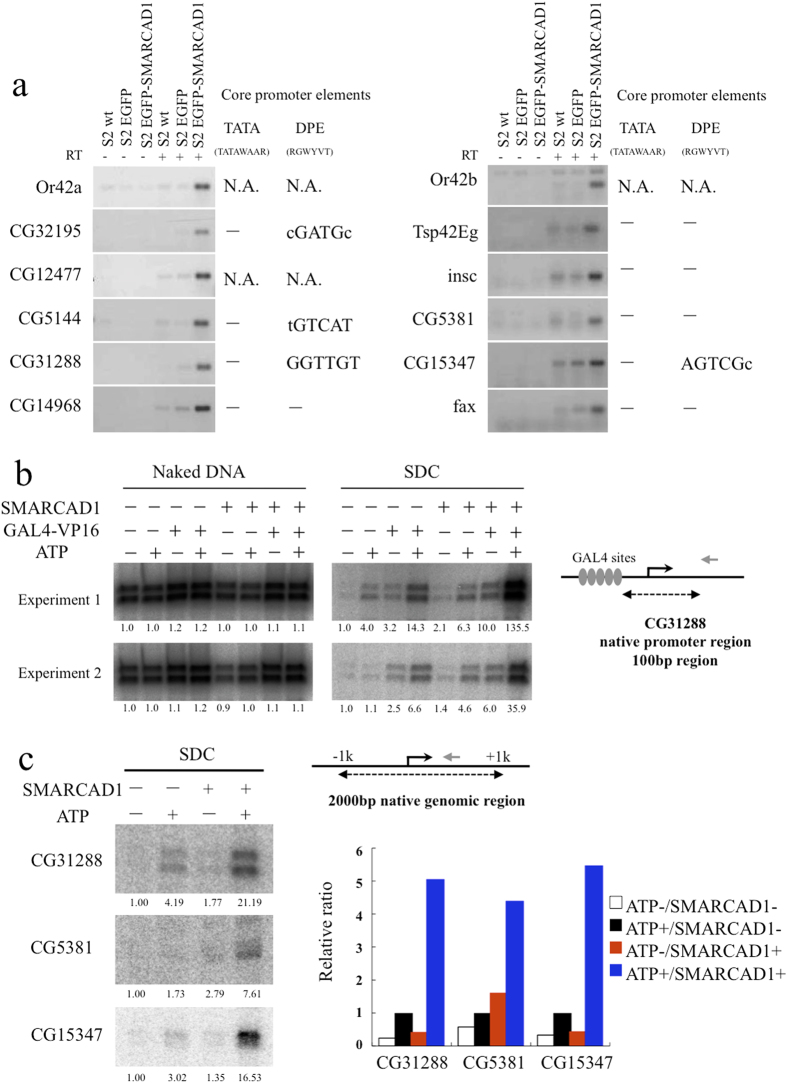
SMARCAD1 promotes transcription in an ATP-dependent manner *in vitro*. (**a**) RT-PCR analyses were performed to confirm changes in gene expression detected by microarray analysis. According to RT-PCR, 12 genes were up-regulated by SMARCAD1 over-expression, which was consistent with microarray analysis. None of the core promoter regions of these genes contain a TATA box, but most promoters had the downstream core promoter element (DPE). N.A. (not assigned); the DNA sequence corresponding to the upstream region of the analyzed gene has not yet been released to the databases. (**b**) *In vitro* transcription using naked DNA (left panels) or SDCs (right panels), which contains the area around the TSS region (from –40 bp–+60 bp) of CG31288 as a template. The reactions were performed in either the presence (+) or absence (–) of the indicated factors. The transcripts were detected by primer extension analysis, and the relative transcript levels are indicated below each band. The scheme at right shows the promoter region of the template DNA, and the gray arrow indicates the primer position. Experiments were carried out in duplicate. (**c**) *In vitro* transcription reactions were carried out with a *Drosophila* nuclear extract with SDCs, which contain the promoter region (from –1 kbp–+1 kbp) of the indicated gene and the indicated factors (left panels). The amount of each signal was normalized by the signal of the ATP (+), SMARCAD1 (–) sample (right panel).

We ectopically expressed EGFP–SMARCAD1 and found up-regulated genes using expression array analysis (Supplementary Fig. 3b [right] and Supplementary table 3). We confirmed 12 up-regulated genes by RT-PCR out of 20 candidate genes detected by expression array analysis (Fig. 4a), suggesting that SMARCAD1 activates transcription of these genes. We further selected one gene, CG31288, joining 100 bp of its core promoter region with five GAL4 binding sites, resulting in an artificial promoter that was subjected to *in vitro* transcription. SMARCAD1 activates transcription together with GAL4-VP16, p300, and general transcription factors in nuclear transcription extracts in an ATP-dependent manner (Fig. 4b). We then selected three genes, CG31288, CG5381, and CG15347, and a 2000-bp genomic region harboring their core promoter that we subjected to chromatin assembly and subsequent *in vitro* transcription. We observed a 7–21-fold transcriptional activation using native genome DNA by SMARCAD1, together with p300 and other factors in nuclear transcription extracts in an ATP-dependent manner (Fig. 4c). These observations strengthen the concept that SMARCAD1 plays a role in transcriptional activation.

### SMARCAD1 activates transcription together with CBP

**Figure 5:**
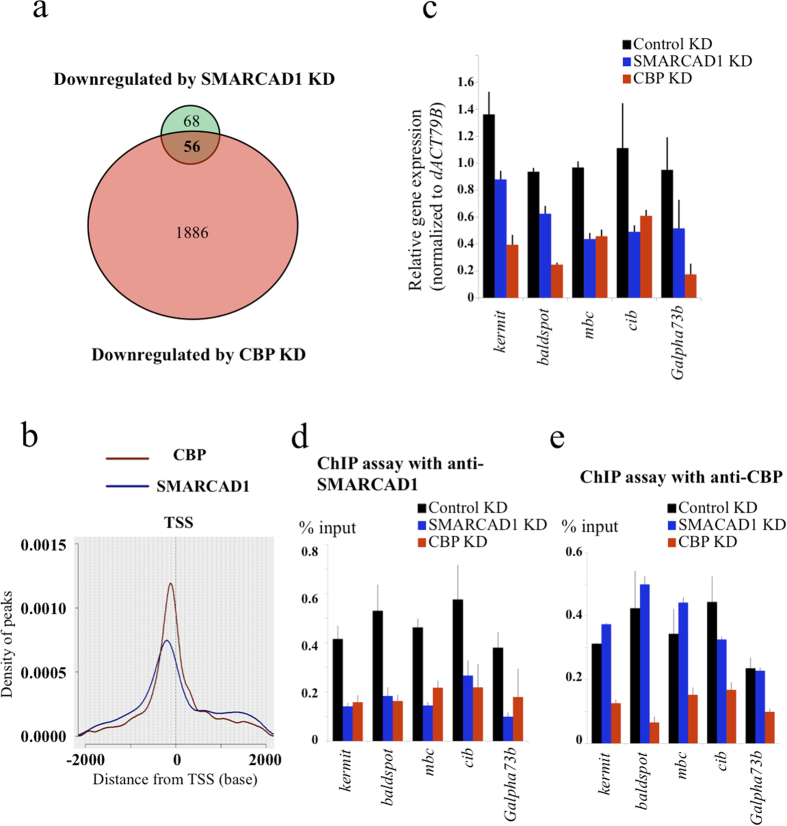
SMARCAD1 is recruited by CBP to the gene promoter and regulates gene expression. (**a**) Venn diagram showing the number of genes down-regulated more than 0.7-fold upon either SMARCAD1 knockdown (KD) or CBP KD relative to control KD. For control KD, nonspecific dsRNA derived from the EGFP sequence was used. (**b**) The distance from the TSS to the promoter targets for CBP and SMARCAD1 is plotted. The Y-axis is the “Density of peaks”, for which the sum is one, and the X-axis indicates “Distance from TSS (bp)”. The TSSs of 3101 genes were defined for SMARCAD1 and that of 3762 genes were defined for CBP based on annotated UCSC coordinates (*D. melanogaster* Genome, dm 6). (**c**) The mRNA levels of five down-regulated genes in both SMARCAD1 KD and CBP KD were quantified by RT-qPCR. Each sample was analyzed by RT-qPCR in technical triplicates and normalized to dACT79B as a control. (**d**,**e**) ChIP-qPCR analysis of SMARCAD1 (**d**) and CBP (**e**) enrichment at the indicated gene promoter. ChIP was performed using anti-SMARCAD1 or anti-CBP antibodies. qPCR following ChIP was performed using TSS-specific primers.

In addition to ectopically expressing SMARCAD1, we knocked down both SMARCAD1 and CBP in S2 cells. First, we determined by RT-qPCR that SMARCAD1 or CBP were knocked down. Knockdown of SMARCAD1 was confirmed by RT-qPCR (Supplementary Fig. 3a). Since knockdown of CBP was unclear following RT-qPCR, we confirmed a decrease in CBP protein by western blotting (Supplementary Fig. 3b). Because acetylation of histones is believed to play a role in transcriptional activation, and, as we found that SMARCAD1 activates transcription *in vitro* (Fig. 4b,c), we focused on the down-regulated genes upon SMARCAD1 and CBP knockdown. However, we also found up-regulated genes caused by SMARCAD1 knockdown. SMARCAD1 is reported to associate with HDAC and KAP1^29^; thus, it can be speculated that these genes might be controlled by the SMARCAD1–HDAC complex, or acetylation by itself might repress transcription directly in some promoters. We detected 124 and 1,942 genes that were down-regulated by at least 0.7 fold by SMARCAD1 and CBP/*nej* knockdown, respectively (Fig. 5a, Supplementary Table 4). Next, we performed ChIP-seq to map SMARCAD1 and CBP-binding sites in S2 cells (Fig. 5b, Supplementary Fig. 4a–d). The genome-wide binding sites of SMARCAD1 and CBP were identified relative to the transcription start site (TSS) (Supplementary Fig. 4a). Moreover, the distance from the ChIP-seq peak to the TSS of the nearest gene showed a similar pattern for CBP and SMARCAD1. These similar distribution patterns suggest cooperation between CBP and SMARCAD1 in the genome. Among 56 overlapping genes in Fig. 5a, we selected five genes from the top of the list and confirmed their regulation by both SMARCAD1 and CBP using qPCR (Fig. 5c). The control ChIP performed using normal rabbit IgG did not show any enrichment at the TSS locus of the same genes (Supplementary Fig. 3c). The signals that remained after knockdown of SMARCAD1 or CBP suggested residual SMARCAD1 or CBP protein after knockdown, based on low signals of the control ChIP experiment using normal rabbit IgG as an antibody control. A ChIP assay with anti-SMARCAD1 antibodies indicated that CBP was required for SMARCAD1 localization to the core promoter region of these genes (Fig. 5d). However, a ChIP assay with anti-CBP antibodies indicated that CBP knockdown only diminishes CBP localization to the core promoter region of these genes (Fig. 5e). These results suggest that CBP initially localizes to the core promoter and that SMARCAD1 is subsequently recruited to the promoter region. Although we generated and purified polyclonal antibodies against acetylated H2A K5 and acetylated H2A K8, these antibodies cross-reacted with acetylated H4 in western blotting (Supplementary Fig. 1c). Therefore, we could not discriminate H2A K5 and K8 acetylation from H4 K5 and K8 acetylation at the TSS of these genes. Hence, we constructed H2A K5A and K8A mutations that do not have a positive charge and overexpressed them in S2 cells. After cloning of the cells expressing H2A mutants, we tested expression of 12 genes that were up-regulated by SMARCAD1 overexpression (Fig. 4a). Interestingly, the CG12477 gene was up-regulated by H2A K5A and K8A mutants in three independent clones (Supplementary Fig. 3d). This result suggests that H2A K5 and K8 acetylation by itself might affect expression of some genes.

**Figure 6:**
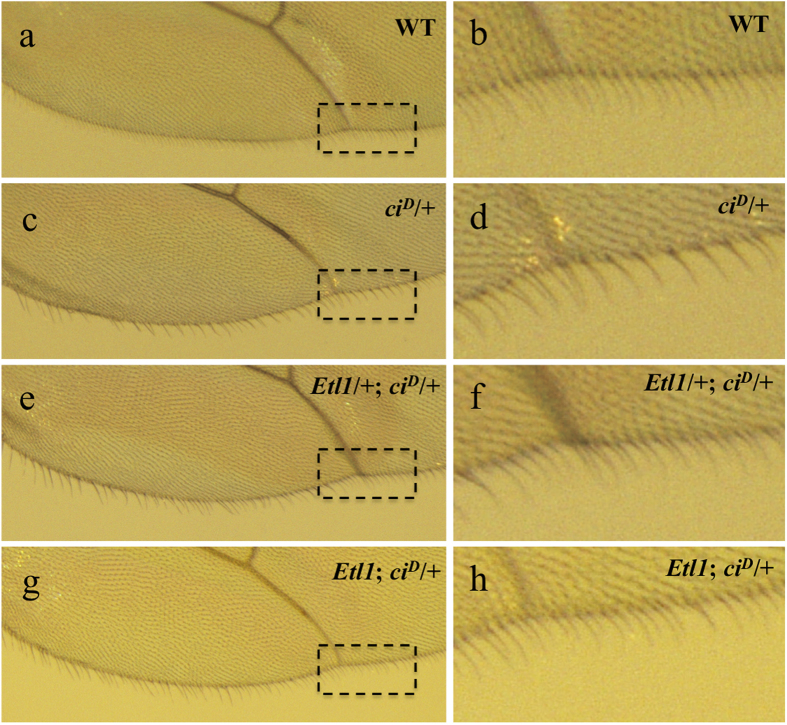
The SMARCAD1/*Etl1* mutation shows a similar posterior-row-hair phenotype as the CBP mutation. Posterior-row-hair phenotypes in wildtype (**a,b**), *ci*^*D*^/+ (**c,d**), *Etl1*/+; *ci*^*D*^/+ (**e,f**), and *Etl1*; *ci*^*D*^/+ (**g,h**) adults. Enlargements of the boxes in (**a,c,e,g**) are shown in (**b,d,f,h**) respectively. *Etl1* represents the homozygous Etl1 mutation, *Etl1/Etl1.*

Next, we examined whether SMARCAD1 functions in a CBP/*nej*-dependent pathway *in vivo*. The cubitus interruptus (ci) gene encodes a transcription factor that is required for induction of hedgehog-dependent transcription, which is an important determinant of pattern formation^30^,^31^. The dominant gain-of-function mutant *ci*^*D*^ causes shortened longitudinal vein 4 of the adult wing, loss of posterior row hairs, and a flattened posterior wing margin. A haploinsufficiency of dCBP suppressed a subset of the *ci*^*D*^ defects. Thus, *Drosophila* CBP/*nej* is a co-activator of *ci* in hedgehog signaling^31^. As previously reported, some posterior row hairs are missing in the dominant gain of function *ci*^*D*^ mutant^31^ (Fig. 6a–d). The *Drosophila* mutation of SMARCAD1 was previously deposited into FlyBase as FBst0017289; P{EP}*Etl1*EP701. The *Etl1* mutant has a p-element insertion in the second intron of the *Etl1* gene. The SMARCAD1/*Etl1* mutation partially suppressed the reduced number of posterior row hairs caused by the *ci*^*D*^ mutation (Fig. 6, compare panel d with panels f and h). This suppression is very similar to that observed when a CBP/*nej*^3^ mutation was present in *ci*^*D*^/+ mutants^31^. The fact that the SMARCAD1/*Etl1* and CBP/*nej*^3^ mutations both suppressed the reduced density of posterior row hairs in *ci*^*D*^/+ mutants suggests that SMARCAD1 and CBP function together in regulating the expression of certain genes.

## Discussion

We demonstrated that SMARCAD1 activates transcription together with p300/CBP *in vivo* and *in vitro*. Although p300/CBP has affinity for nucleosomes^32^, once a histone octamer is wrapped with DNA, it becomes hard for histone modification enzymes to access core histones, as we have shown in Fig. 2e, lanes 2 and 6. We suggest that SMARCAD1 facilitates p300/CBP access to core histone tails and facilitates core histone acetylation. Previously, it has been shown that ISWI-containing complexes, such as NURF or ACF, facilitate core histone acetylation and transcription using an artificial promoter^24^,^33^. Using a native promoter in this work, we demonstrated that, together with p300/CBP, ATP-dependent SMARCAD1 activity plays a role in histone H2A acetylation, resulting in up-regulation of the target genes. Furthermore, similar distances from the TSS to the CBP- or SMARCAD1-targeted promoters suggest that CBP and SMARCAD1 work together (Fig. 5b). From the overlap of gene sets in Fig. 5a, we have demonstrated that CBP assembles on the promoter first and subsequently recruits SMARCAD1 before transcription (Fig. 5d,e). Together with previous reports, we suggest that diverse remodeling activities work together with p300/CBP at different promoters.

CBP/p300 acetylates numerous proteins, including all core histones. K5 of H2A can be acetylated in bulk chromatin by p300 *in vivo*, but K8 was not known to be acetylated^34^. Here, we found that K8 of H2A was a new target of acetylation by CBP *in vitro*. We confirmed that H2AK8 and H2AK5 are acetylated in *Drosophila* embryos using western blotting (Supplementary Fig. 1c). It is likely that the acetylation of H2AK8 is a novel histone mark *in vivo*, and this modification might play a role in transcriptional regulation together with the acetylation of H2AK5 in *Drosophila*.

There are two *Drosophila* H2A proteins, canonical H2A and variant H2A.V. Having an amino acid sequence homology of 55% with H2A, H2A.V contains the H2A.X phosphorylation site at the C-terminus^35^. H2A.V becomes phosphorylated at DNA double-strand breaks (DSBs), and the dTip60 chromatin-remodeling complex preferentially acetylates phospho-H2A.V and converts it to an unmodified H2A.V during DSB repair^36^. Although we performed a HAT assay using chromatin assembled with plasmid DNA and recombinant canonical core histones, it will be important in the future to determine whether variant H2A.V is acetylated at the conserved N-terminal lysine by CBP in cooperation with SMARCAD1.

SMARCAD1 belongs to the Swr1-like grouping, as do Swr1 and Ino80. The Swr1 protein is a part of the SWR1 complex, which replaces canonical H2A–H2B dimers with histone variant H2A.Z–H2B dimers in an ATP-dependent manner^37^. The incorporation of the H2A.Z-containing nucleosome at gene promoters by the SWR1 complex was involved in their transcriptional activation. Similarly, Ino80 forms a complex, which is known as Ino80.com, several subunits of which are common to the SWR1 complex. Ino80 is recruited to chromosomal DSBs and plays a critical role in the eviction of H2A.Z, γH2A.X, and core histones located near the breaks^38^. Furthermore, Ino80 and Swr1 compete for the incorporation of H2A variants during DNA damage^39^. It would be interesting to find out the fate of the acetylated H2A in the nucleosome, such as eviction or exchange, when acetylated by CBP in cooperation with SMARCAD1.

SMARCAD1 has been previously shown to be important for maintenance of silent chromatin throughout replication and to associate with HDAC1/2, KAP1, histones, and the methyltransferase G9a/GLP, playing a role in ensuring that silenced loci are correctly perpetuated^29^,^40^. Human SMARCAD1 occurs in two isoforms: the large isoform, which is expressed ubiquitously, and the short isoform, which is mainly expressed in skin fibroblasts^41^. A heterozygous splicing mutation in a short isoform of human SMARCAD1 can cause adermatoglyphia (loss of fingerprints) and Basan syndrome, an ectodermal dysplasia with autosomal dominant inheritance and intra- and interfamilial variability^42^. Furthermore, SMARCAD1 and Fun30, the yeast homologue of human SMARCAD1, were found to have a conserved role in controlling homologous recombination and genome stability^43^. A previous study reported that Fun30 is required for silencing by regulating the chromatin structure within transcriptionally repressed domains^44^. These observations suggest multiple roles for SMARCAD1 in maintaining chromatin integrity not only for transcriptional activation or repression but also for DNA repair.

We propose that chromatin remodeling by SMARCAD1 plays a role in chromatin repression together with repressing complex, in transcriptional activation together with co-activators such as p300/CBP, and in DNA repair and genome stability with different partners. The multifaceted functions of SMARCAD1 are determined by versatile partners and result in the maintenance of chromatin and the genome-integrity network.

## Materials and Methods

### Ethics statement

All recombinant DNA experiments in this study strictly followed a protocol and guidelines approved by the Institutional Recombinant DNA Experiment Safety Committee of Nagasaki University (approval number: 1503121303).

### Purification of native SMARCAD1

*Drosophila* SMARCAD1 was purified from a soluble nuclear extract prepared from 0–12-h *Drosophila* embryos. Nuclear extracts were prepared as previously described^45^ and subjected to sequential chromatographic steps. In all column chromatography steps, samples were applied to the column and washed with 10 column volumes of 0.1 M KCl in HEG buffer (25 mM HEPES at pH 7.6, 0.1 mM EDTA, 10% glycerol, 0.01% NP40, 1 mM benzamidine, 0.2 mM PMSF). Binding proteins were eluted with 10 column volumes of a linear gradient, from 0.1–1.0 M KCl, in HEG buffer. All fractions of each chromatographic step were analyzed by HAT assay, and the fractions that contained ATP-dependent HAT activity were loaded onto the next column.

First, the nuclear extracts from 1 kg of embryos were applied to a 120-ml Q-Sepharose FF (GE Healthcare) column. HAT activity was mainly eluted in 0.4-M KCl fractions. This Q-Sepharose step was repeated three more times, and the 0.4-M KCl fractions that contained the HAT activity were pooled. Second, these fractions were diluted to 0.1 M KCl and applied to a 30-ml SP-Sepharose FF (GE Healthcare) column. HAT activity was mainly eluted in the 0.6-M KCl fractions. Third, these fractions were diluted to 0.1 M KCl and applied to an 8-ml POROS heparin (Roche) column. HAT activity was mainly eluted in the 0.7-M KCl fractions. Fourth, these fractions were diluted to 0.1 M KCl and applied to a 0.5-ml Blue Sepharose FF (GE Healthcare) column. HAT activity was mainly eluted in the 0.7-M KCl fractions. Fifth, these fractions were diluted to 0.1 M KCl and applied to a 0.1-ml Mono S (GE Healthcare) column. HAT activity was mainly eluted in the 0.7-M KCl fractions. To identify the proteins responsible for HAT activity, the HAT-positive gel fractions were excised from the gel, from top to bottom, digested with trypsin, and subsequently analyzed by LC–MS/MS as described previously^46^.

### Plasmid construction

pAc–EGFP–Flag–SMARCAD1 was constructed by inserting EGFP–Flag– SMARCAD1 cDNA into the pAc5.1/V5-HisA vector (Life Technologies) for expression in S2 cells. pFastBac1–Flag–SMARCAD1 was constructed by inserting Flag-tagged SMARCAD1 cDNA into the pFastBac1 vector for expression in sf9 cells using the BAC-TO-BAC baculovirus expression system (Life Technologies). For *in vitro* transcription, a template plasmid was constructed by subcloning the fragment from –40 bp–+60 bp relative to the transcription start site (TSS) of the CG31288 gene and fragments from the –1 Kbp–+1 Kbp region relative to the TSS of CG31288, CG5381, and CG15347 genes into pGIE0, downstream of the five GAL4-binding sequences and upstream of the AdE4 promoter, resulting in pTXCG31288P, pTXCG31288G, pTXCG5381G, and pTXCG15347G.

### Generation of antibodies

Rabbit anti-SMARCAD1 antibodies and anti-CBP antibodies ware generated against recombinant proteins for full-length *Drosophila* SMARCAD1 and partial *Drosophila* CBP (residues 1550–2143), respectively, and the antisera were collected and purified using Affi-Gel 10 (BIO-RAD) on a column made with recombinant SMARCAD1 or recombinant CBP, as described previously^47^. Rabbits were immunized with the peptides for *Drosophila* H2A (acetyl-K5) or H2A (acetyl-K8), and then antisera were collected, absorbed with non-acetylated H2A peptide, and purified with H2A (acetylated K5) or (acetylated K8) peptides using SulfoLink Coupling Gel (Thermo Fisher Scientific), respectively.

### Purification of recombinant protein

Flag-tagged SMARCAD1 was purified from baculovirus-infected Sf9 cells by three conventional chromatographic steps: SP-Sepharose FF (GE Healthcare), Q-Sepharose FF (GE Healthcare), and Toyopearl heparin (Tosoh). The peak fraction of the final Toyopearl heparin column in which Flag-tagged SMARCAD1 was expressed was immunopurified with ANTI-FLAG M2 agarose (Sigma-Aldrich). Flag-tagged SMARCAD1 was eluted with Flag peptides (Sigma-Aldrich) and dialyzed against 100 mM KCl buffer. Recombinant p300 was prepared as described previously.

### Histone acetyltransferase assay

Chromatin was reconstituted with plasmid and purified *Drosophila* core histones using salt dialysis techniques. For the HAT assay, recombinant SMARCAD1 was incubated with 75 ng of chromatin as substrate in the presence of 1 μM [^3^H]-acetyl-CoA, cold 10 μM acetyl-CoA, recombinant p300, 10 mM sodium butyrate, 3 mM ATP, 5 mM MgCl_2_, and 50 nM GAL4-VP16 for 3 h at 27 °C. Reaction mixtures were separated on 13% SDS-PAGE and treated with Enlightning radiography enhancer (PerkinElmer), dried, and exposed to X-ray film.

### Immunodepletion

Antibodies were incubated for 4 h with protein A–Sepharose beads (GE Healthcare), followed by three washings in 0.5 M KCl buffer and 0.2 M borate buffer and resuspension in 1 ml of 0.2 M borate buffer with dimethyl pimelimidate. The slurry was incubated for 30 min at room temperature (RT), sedimented, and further incubated with 0.2 M ethanolamine for 2 h at RT. The beads were then incubated for 4 h at 4 °C in an SP column fraction after Q Sepharose treatment of the nuclear extract, and the supernatant was then assayed for HAT activity.

### Determination of the acetylation site

Salt-dialyzed chromatins (SDCs) were assembled with plasmid DNA, recombinant histones, the active fraction resulting from POROS heparin chromatography, and [^3^H]-acetyl-CoA. After acetylation, the reaction mixture was subjected to electrophoresis on 13% SDS-PAGE and transferred to a PVDF membrane. After Coomassie Brilliant Blue (CBB) staining, the bands corresponding to H2A/H2B were excised and subjected to automated Edman sequencing. [^3^H]-radioactivity eluted from each cycle was monitored with a liquid scintillation counter.

### Immunostaining of *Drosophila* embryos

*Drosophila* embryos were stained, essentially as previously described^23^. Basically, 0–1-h *Drosophila* embryos were collected and washed with cold water. The chorions were removed by breech, and the remaining embryos were suspended in buffer containing 70 mM NaCl and 0.03% Triton X-100. After devitalizing, embryos were then incubated four times for 30 min each in 10% bovine serum albumin in PBS. Subsequently, the embryos were incubated with Alexa 488-labeled anti-SMARCAD1 antibodies that had been labeled overnight at 4 °C using a Zenon Rabbit IgG labeling kit (Molecular Probes). The embryos were washed and then incubated with Hoechst 33342 dye for visualizing cellular DNA. The embryos were washed, and confocal images were collected with an Olympus IX81 microscope.

### RNA extraction from *Drosophila*

The staged embryos were collected and dechorionized as described above. Larvae were collected and frozen in liquid nitrogen. Total RNA was extracted using ISOGEN II reagent (Nippon Gene), according to the manufacturer’s instructions.

### Microarray

Total RNA (300 ng) was amplified and biotin labeled with the GeneChip 3′ IVT Express Kit (Affymetrix) according to the manufacturers’ instructions. The biotinylated antisense cRNAs were hydrolyzed and hybridized to the GeneChip (R) *Drosophila* Genome 2.0 Array (Affymetrix) for 16 h at 45 °C. Washes and the subsequent antibody hybridization process were performed using the Fluidics Station 450 (Affymetrix) according to the manufacturers’ instructions, and the arrays were scanned using a GeneChip Scanner 3000 (Affymetrix). Microarray data were summarized using the Robust Multichip Average (RMA) method.

### *In vitro* transcription reaction

Salt-dialyzed chromatins (SDCs) were prepared as described previously^48^. The modified pGIE0 template containing five GAL4 binding sites upstream of the genome sequence of interest was incubated with purified *Drosophila* core histones. The SDCs were pre-incubated with 10 mM sodium butyrate, 5 mM MgCl_2_, 0.1 mM acetyl-CoA, 3 mM ATP, and SMARCAD1 before transcription, and the transcription reaction was carried out with nuclear extract from *Drosophila* embryos. For transcription using pGIE0 plasmid DNA, which has GAL4 binding sites upstream of the AdE4 promoter, and using pTXCG31288P, GAL4-VP16 was added as indicated. For transcription using pTXCG31288G, pTXCG5381G, and pTXCG15347G, GAL4-VP16 was not added. Using pTXCG31288G, pTXCG15347G, and pTXCG5381G, transcripts were detected from the native promoter but not from the adenovirus E4 promoter. The resulting transcripts were detected by primer extension using ^32^P-labeled primers, which were designed against a sequence located approximately 87 bp downstream of the transcription start sites. The reverse-transcribed DNA products were separated with an 8% urea/acrylamide gel and visualized by autoradiography.

### Cell culture and transfections

S2 cells were grown at 26 °C in Schneider’s *Drosophila* medium (Gibco), supplemented with 10% FBS. S2 cells were transfected using Lipofectin reagent (Invitrogen), according to the manufacturers’ recommendations. In brief, to obtain a stable clone, pAc–EGFP–Flag–SMARCAD1 or pAc–EGFP was co-transfected with pCoHygro (Invitrogen) using Lipofectin reagent. After 22 h of transfection, DNA-containing medium was replaced with selection medium supplemented with 10% FBS and 300 μg/mL HygroGold (InvivoGen).

### RNA interference in *Drosophila* S2 cells

Double-strand RNAs (dsRNAs) were generated using the MEGAscript T7 kit (Ambion). The primer sequences for generation of SMARCAD1, CBP, and enhanced green fluorescent protein (EGFP), as a control, were made using a dsRNA template with PCR as follows:

SMARCAD1 forward,

5′-TAATACGACTCACTATAGGGTACAGTCGGACAGCACAGTG-3′;

SMARCAD1 reverse,

5′-TAATACGACTCACTATAGGGTACGACTGGAGTTCAATTAGCGAAGC-3′;

CBP forward,

5′-TAATACGACTCACTATAGGGTACATGGCCGATCACTTAGAC-3′;

CBP reverse,

5′-TAATACGACTCACTATAGGGTACATGGGCATTGAGTTGACC-3′;

EGFP Forward,

5′- TAATACGACTCACTATAGGGTACATGGTGAGCAAGGGCGAGGAG -3′;

and EGFP Reverse,

5′- TAATACGACTCACTATAGGGTACTTACTTGTACAGCTCGTCCAT -3′.

S2 cells (2 × 10^7^) were plated in 5 ml of 1× Schneider’s *Drosophila* medium without FBS in 10-cm dishes. dsRNA was added at 26 °C for 6 h, followed by 5 ml of 1× Schneider’s *Drosophila* medium with 10% FBS. The cells were then incubated for an additional 72 h to allow for turnover of the target mRNA.

### Quantitative RT-PCR analysis

Total RNA was extracted from S2 cells treated with transfection or RNAi using ISOGEN II reagent. Reverse transcription was performed with M-MuLV reverse transcriptase (NEB), random hexamers (Takara), and oligo(dT) primers (Invitrogen). Quantitative RT-PCR was performed using the KAPA SYBR FAST qPCR kit (Kapa Biosystems). *dACT79B* was used as an internal control.

### ChIP assay

For ChIP analysis, S2 cells were cross-linked by adding 1% formaldehyde to cells for 10 min at 26 °C and then washed in cold PBS. After washing with solution I (10 mM HEPES at pH 7.5, 10 mM EDTA, 0.5 mM EGTA, and 0.75% Triton X-100) and solution II (10 mM HEPES at pH 7.5, 200 mM NaCl, 1 mM EDTA, and 0.5 mM EGTA), the pellet was resuspended in MNase buffer (15 mM Tris-HCl at pH 7.5, 5 mM MgCl_2_, 1 mM CaCl_2_, and 25 mM NaCl). Subsequently, samples were incubated with MNase (Sigma-Aldrich) for 10 min at RT, and then EDTA and lysis buffer were added (150 mM NaCl, 25 mM Tris at pH 7.5, 5 mM EDTA, 1% Triton X-100, 0.1% SDS, and 0.5% deoxycholate [DOC]). Samples were sonicated extensively (20% output, 2 × 12 sec) and centrifuged at 14,000 rpm for 10 min at 4 °C. The supernatant was used for ChIP, and chromatin was immunoprecipitated overnight at 4 °C with the following antibodies: anti-SMARCAD1 and anti-CBP, together with normal rabbit IgG as a control. Bound DNA was precipitated by protein A–Sepharose (GE Healthcare). The immunoprecipitates were washed with the following combination of wash buffers: RIPA buffer (50 mM Tris at pH 8.0, 1 mM EDTA, 150 mM NaCl, 1% NP40, 0.1% SDS, and 0.5% DOC), high salt buffer (50 mM Tris at pH 8.0, 1 mM EDTA, 500 mM NaCl, 1% NP40, 0.1% SDS, and 0.5% DOC), LiCl buffer (50 mM Tris at pH 8.0, 1 mM EDTA, 250 mM LiCl, 1% NP40, and 0.5% DOC), and TE buffer. Bound chromatin and input DNA were treated with RNase A (Nacalai Tesque) at 37 °C for 30 min. For reverse cross-linking, the beads were incubated at 37 °C for 6 h followed by 65 °C for 12 h with TE buffer containing 0.5% SDS and 250 μg/ml proteinase K (Roche) and then purified by phenol/chloroform extraction. The resulting immunoprecipitated DNA was analyzed with qPCR for quantification. qPCR primers are listed in Supplementary Table 2.

### ChIP-seq

ChIP-seq libraries were prepared using TruSeq ChIP Sample Prep Kit (Illumina) according to the manufacturers’ instructions. Libraries ware sequenced with 76-bp paired-end read sequencing on the MiSeq II sequencer (Illumina).

### ChIP-seq analysis

Sequenced reads were mapped to the *Drosophila* genome (UCSC genome browser assembly dm 6, BDGP Release 6) using the Burrows-Wheeler Alignment v0.7.5 (BWA) program^49^, with default “aln” and “samse” parameters. We used the Model-based Alignment of ChIP-Seq (MACS) program^50^ to call peaks for SMARCAD1 and CBP. The following parameters were used: macs; t, treatment_bamfile; c, control_bamfile; bdg; P = 0.05.

After identification of SMARCAD1 and CBP peak regions, annotation of these peaks was performed using the R package ChIPpeakAnno^51^. This software partitioned the genome into six discrete regions based on annotated UCSC coordinates: (1) Downstream, downstream of the TES; (2) IncludeFeature, contains gene-coding sequence; (3) Inside, intergenic; (4) OverlapEnd, overlapping with the TES; (5) OverlapStart, overlapping with the TSS; (6) Upstream, upstream of the TSS. The software then output the localization attributes of the nearest features according to the partitions.

### Drosophila stocks

The fly line for *Etl1* [EP701] was obtained from the Bloomington *Drosophila* Stock Center (Indiana University).

## Additional Information

**Accession code**: The accession number for the ChIP-seq data reported in this paper is GEO: GSE72666.

**How to cite this article**: Doiguchi, M. *et al.* SMARCAD1 is an ATP-dependent stimulator of nucleosomal H2A acetylation via CBP, resulting in transcriptional regulation. *Sci. Rep.* **6**, 20179; doi: 10.1038/srep20179 (2016).

## Supplementary Material

Supplementary Information

Supplementary Table 3

Supplementary Table 4
