# Supplementary material for: SMARCAD1 is an ATP-dependent stimulator of nucleosomal H2A acetylation via CBP, resulting in transcriptional regulation
Source: Sci Rep. 2016 Feb 18;6:20179. doi: 10.1038/srep20179 (PMC4757861; doi:10.1038/srep20179)
Supplement: Supplementary Information [file srep20179-s1.pdf]

SMARCAD1 is an ATP-dependent stimulator of nucleosomal H2A acetylation via CBP, resulting in transcriptional regulation

[*Keywords*: acetylation; CBP; histone modification; SMARCAD1; H2A]

Running Head:

SMARCAD1 and CBP Activate Transcription

Masamichi Doiguchi[1, 8], Takeya Nakagawa[1, 8], Yuko Imamura[1], Mitsuhiro Yoneda[1], Miki Higashi[1], Kazuishi Kubota[2], Satoshi Yamashita[3], Hiroshi Asahara[3], Midori Iida[4], Satoshi Fujii[4], Tsuyoshi Ikura[5], Ziying Liu[6], Tulip Nandu[6], W. Lee Kraus[6], Hitoshi Ueda[7], Takashi Ito[1, 9]

[1]Nagasaki University School of Medicine, Nagasaki 852-8523, Japan.

[2]Daiichi Sankyo RD Novare CO., LTD., Tokyo 134-8630, JAPAN

[3]Tokyo Medical and Dental University, Tokyo 113-8510, Japan

[4] Kyushu Institute of Technology, Fukuoka820-8502, Japan

[5]Radiation Biology Center, Kyoto University, Kyoto 606-8501, Japan.

[6]UT Southwestern Medical Center, Dallas, TX, 75390, USA

[7]Okayama University, Okayama 700-8530, Japan.

[8]These authors contributed equally to this work..

[9]Corresponding author.

E-mail: tito@nagasaki-u.ac.jp, Tel: 81-95-819-7037, Fax: 81-95-819-7040

**Name**	**sequence**	**score**	**Mr (expt)**
nej	TALLPTLEK	29	984.60
	LGFDIDDGSALADHK	17	1572.77
Topoisomerase II	ISNYNPR	49	862.44
	AYDVAASSK	26	910.46
	LSELESSR	8	945.54
	ITFSPDLAK	29	990.56
	AEEQGINLK	49	1000.54
	IVHEVANER	24	1065.59
	DFNGTDYTR	44	1087.48
	EYFQDMDR	16	1102.46
	NTDDDSGPPIK	41	1157.52
	WEVACCPSDR	31	1278.53
	ENVLEPLSNGTEK	16	1428.72
POLO	VPSYLR	26	733.40
	LLSYFK	12	769.42
	STDIPDR	36	802.38
	IGDFGLATR	55	948.50
	SITEFECR	23	1040.44
	TAQEITIHR	22	1067.55
	MSAITYMDQEK	13	1315.53
	IGSNDTIEDSMHR	21	1473.60
	AGANNVNIESDQISR	29	1586.70
	AGANNVNIESDQISR	(9)	1586.72
belle	NNVALAR	36	756.43
	VGLENIR	20	799.47
	YDKPTPVQK	14	1074.59
	FLVLDEADR	46	1076.55
	ELATQIFEEAK	25	1277.65
	QSGDYGYGSGGGGR	33	1316.54
	ELATQIFEEAKK	8	1405.74
	WKEGGGSNVDYTK	20	1439.65
	GGGGGSGSNLNEQTAEDGOAQQQQQP R	39	2698.16
SMARCAD1	SSLSDLR	30	776.42
	LDGATAVNVR	71	1014.56
	WCPELVVEK	9	1158.58
	IPDNLICDSGK	26	1230.60
	AISNGAGIVEQPK	58	1282.69
Gck III	INTIMDR	41	861.44
	SVFELAER	16	949.50
	AANVLLSEQGDVK	18	1342.71

qPCR primers

RT-qPCR	SMARCAD1	Forward	TAAGAACCTGCCCAAGAAGC
		Reverse	TTCGCTGCTGCTGCACACCTC
	CBP	Forward	TGTTGCTATCACGCGAAGAAC
		Reverse	TGAACGCGACATGAGCGCCAC
	ACT79B	Forward	GCCTCCGGCCGTACCACCGG
		Reverse	CGGCCAGATCTAGACGAAGG
	kermit	Forward	CAGCGCGTCATCAGCACAG
		Reverse	TGGGATTGGATAGTGTCCTCG
	baldspot	Forward	CTGGACCTGGCTCTTCGTGC
		Reverse	CCGTATAGCTGAACCACGAG
	mbc	Forward	CTCAGTCTAAGATGGGATGC
		Reverse	TCATGATGTTGATGCACTCG
	cib	Forward	TAGTAGTCCGCTTCGCCAATC
		Reverse	ACTGGCTTTTCAGGTTCTCG
	Galpha73b	Forward	GTACTGCGACCACGTCACTAC
		Reverse	GCACAAAGTTGTCCAGGAAG
ChIP-qPCR	kermit	Forward	CTCAATAGGCCGATTGTCAGC
		Reverse	AATTCGGACCTCGCGCTGTAG
	baldspot	Forward	TACCGGGATGGCAAAAGTACC
		Reverse	TTCTGCCCCAGCGACTCGG
	mbc	Forward	CCCATTTGACATCAACGGTGTG
		Reverse	CCGCTCTCGGAAAACAACCCG
	cib	Forward	GCATGGTGTTTGTATCAGCTAG
		Reverse	GGCACAGTCCATTTAGTGGAG
	Galpha73b	Forward	CCACTCGATGGAGCTCTCTC
		Reverse	CCTGCCGGAATACGTCTAAC

Supplementary Fig. 1

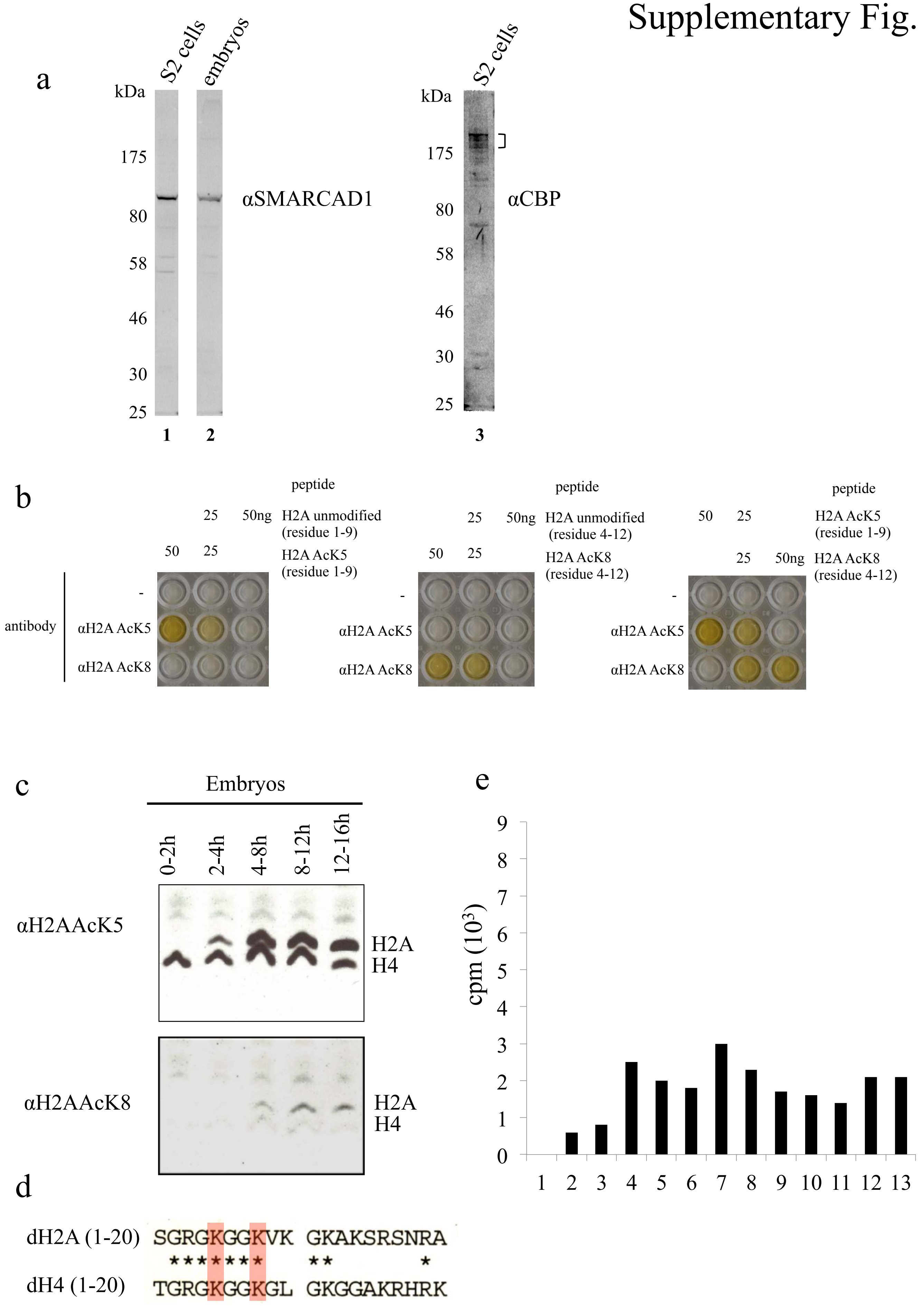

Supplementary Fig. 2a

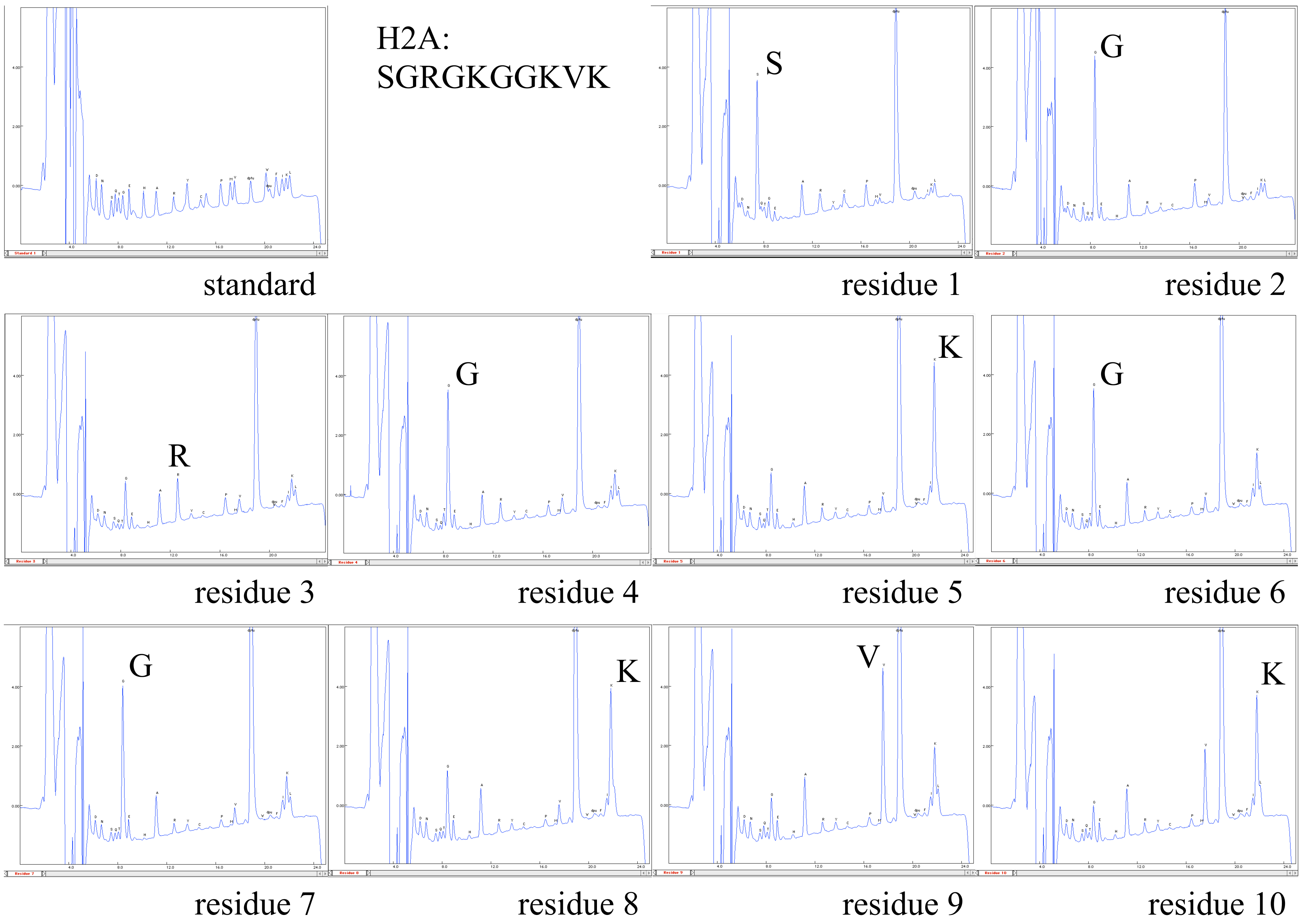

Supplementary Fig. 2b

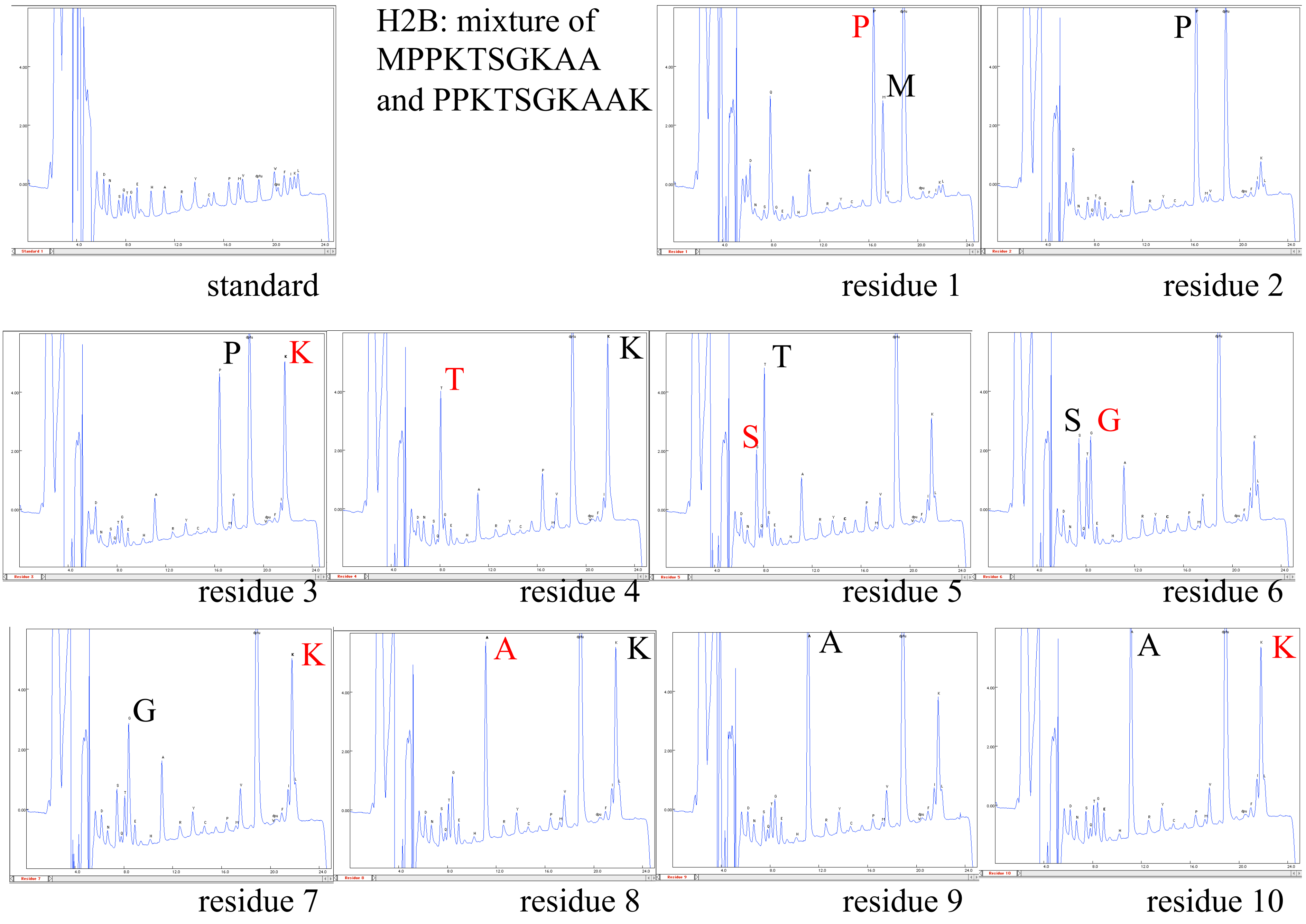

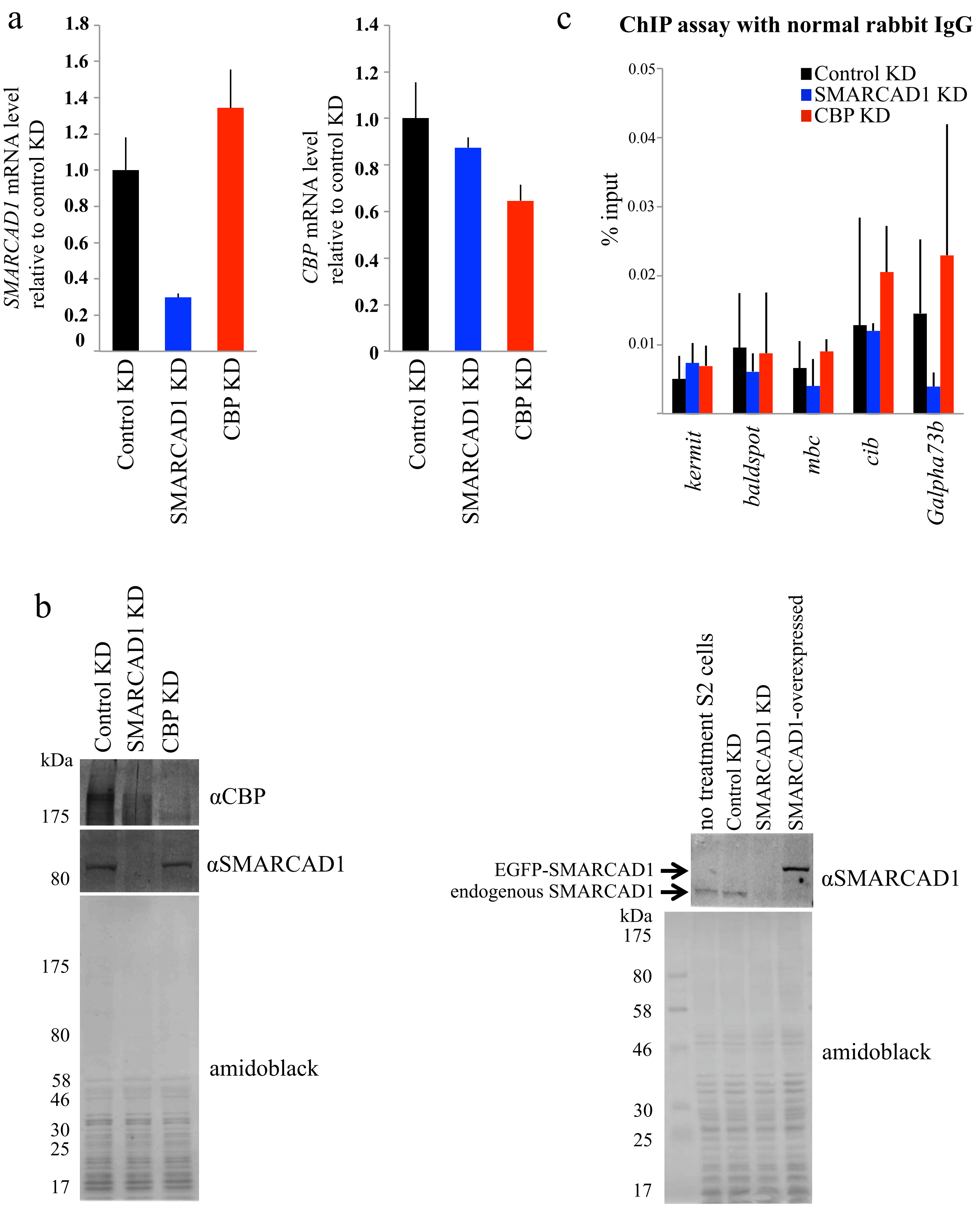
a
SMARCAD1 mRNA level relative to control KD
CBP mRNA level relative to control KD
Control KD
SMARCAD1 KD
CBP KD
c
ChIP assay with normal rabbit IgG
% input
Control KD
SMARCAD1 KD
CBP KD
kermit
baldspot
mbc
cib
Galpha73b
b
kDa
αCBP
αSMARCAD1
amidoblack
no treatment S2 cells
SMARCAD1-overexpressed
EGFP-SMARCAD1
endogenous SMARCAD1

d

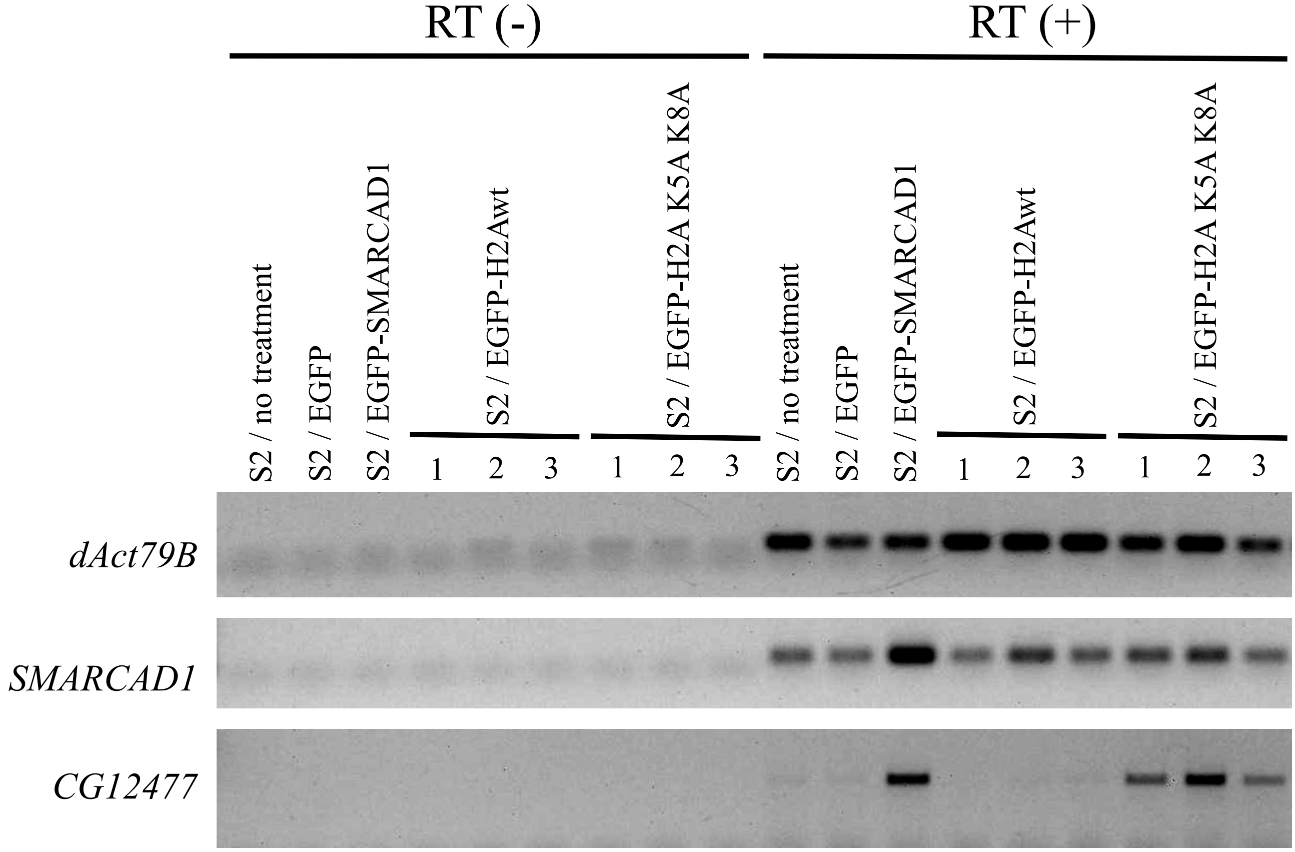
RT (-)
RT (+)
S2 / no treatment
S2 / EGFP
S2 / EGFP-SMARCAD1
S2 / EGFP-H2Awt
S2 / EGFP-H2A K5A K8A
1
2
3
dAct79B
SMARCAD1
CG12477

Supplementary Fig. 4

a

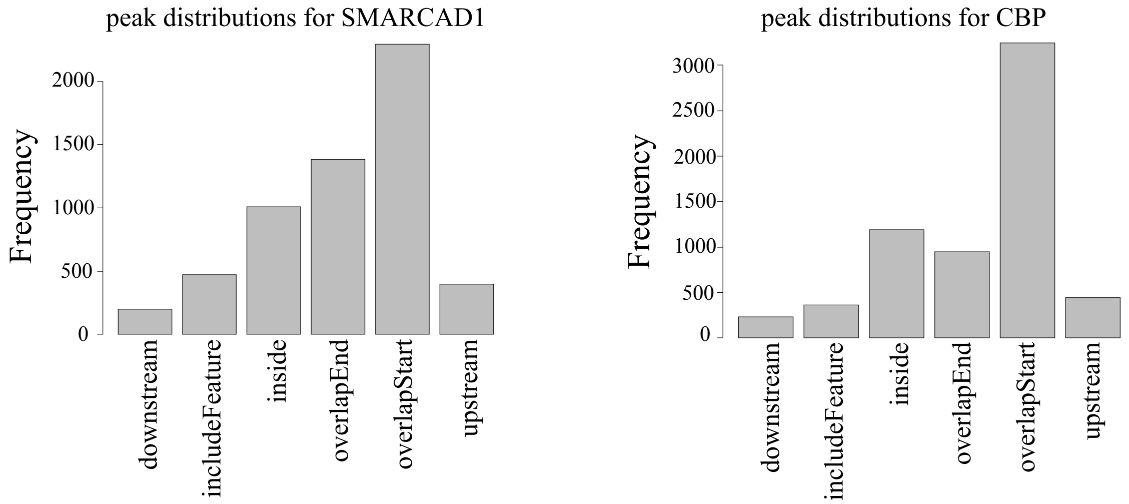

	SMARCAD1<br># of peaks	CBP<br># of peaks
downstream	199 (4%)	230 (4 %)
includeFeature	471 (8%)	363 (6 %)
inside	1007 (18%)	1191 (19%)
overlapEnd	1383 (24%)	949 (15%)
overlapStart	2292 (40%)	3243 (50%)
upstream	397 (7%)	442(7%)

b

Annotation Cluster 1 (Enrichment Score: 4.91)

GO ID	Term	Count	P Value	FDR
GO:0032553	ribonucleotide binding	232	5.6E-06	0.01
GO:0032555	purine ribonucleotide binding	232	5.6E-06	0.01
GO:0000166	nucleotide binding	292	7.6E-06	0.00
GO:0017076	purine nucleotide binding	243	1.8E-05	0.01
GO:0005524	ATP binding	185	1.6E-04	0.03
GO:0032559	adenyl ribonucleotide binding	185	1.8E-04	0.03
GO:0030554	adenyl nucleotide binding	196	4.0E-04	0.05
GO:0001883	purine nucleoside binding	197	4.1E-04	0.04
GO:0001882	nucleoside binding	197	6.6E-04	0.06

Annotation Cluster 2 (Enrichment Score: 4.5)

Category	Term	Count	PValue	FDR
GO:0007010	cytoskeleton organization	136	1.80E-09	0.00
GO:0000226	microtubule cytoskeleton organization	89	6.30E-07	0.00
GO:0007052	mitotic spindle organization	64	7.70E-07	0.00
GO:0007017	microtubule-based process	111	2.80E-06	0.00
GO:0000278	mitotic cell cycle	99	6.20E-06	0.00
GO:0007051	spindle organization	68	1.00E-05	0.00
GO:0007049	cell cycle	142	1.00E-03	0.07
GO:0022402	cell cycle process	125	3.00E-03	0.11
GO:0022403	cell cycle phase	107	3.10E-02	0.41
GO:0000279	M phase	101	5.80E-02	0.52

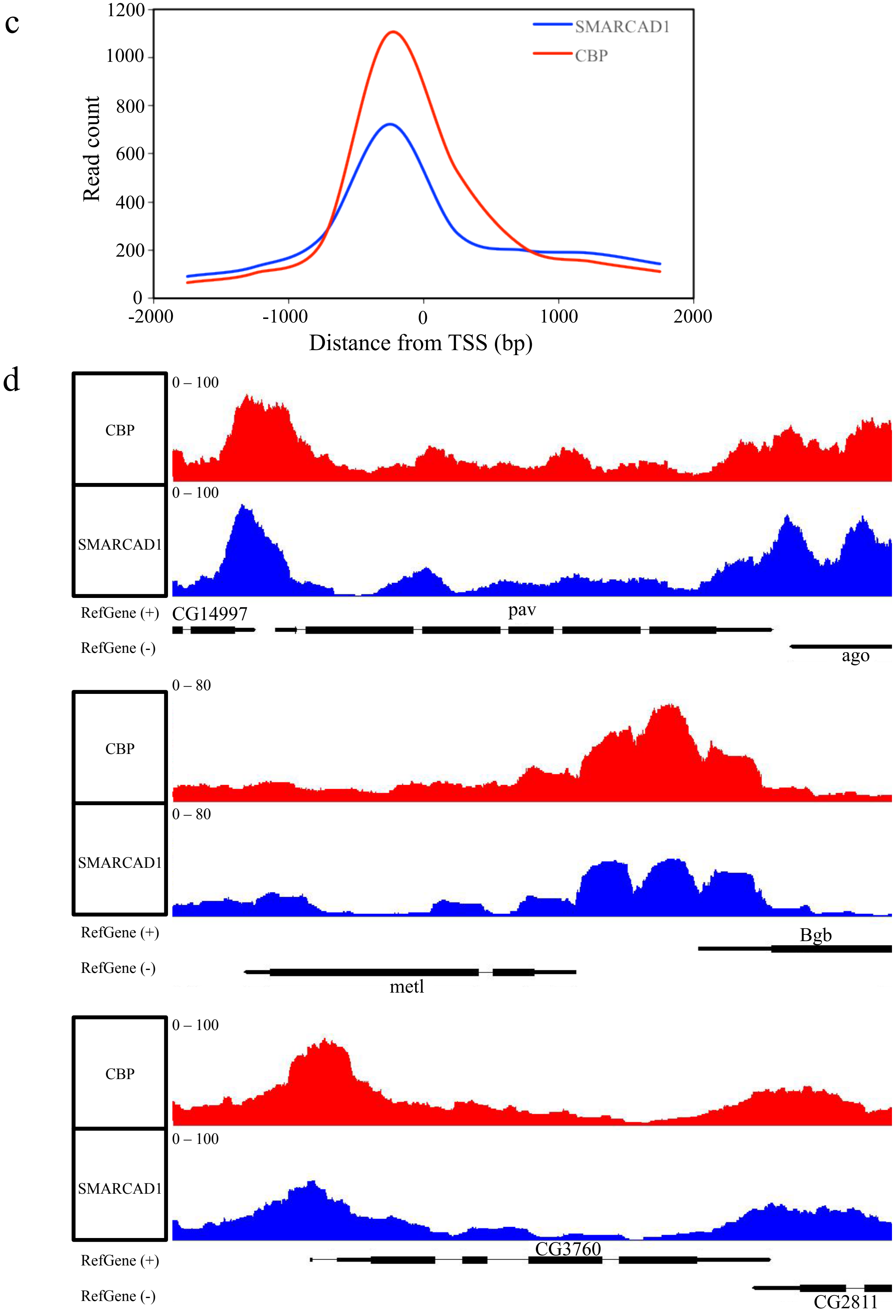
c
1200
1000
800
600
400
200
0
Read count
SMARCAD1
CBP
-2000
-1000
0
1000
2000
Distance from TSS (bp)
d
CBP
0 – 100
SMARCAD1
0 – 100
RefGene (+)
CG14997
pav
RefGene (-)
ago
CBP
0 – 80
SMARCAD1
0 – 80
RefGene (+)
Bgb
RefGene (-)
metl
CBP
0 – 100
SMARCAD1
0 – 100
RefGene (+)
CG3760
RefGene (-)
CG2811

Supplementary information

Supplementary methods

Plasmid construction

pAc–EGFP–Flag–H2A(wt) and pAc–EGFP–Flag–H2A(K5A, K8A) were constructed by inserting EGFP–Flag–H2A and –H2A(K5A, K8A) mutant cDNA into the pAc5.1/V5-HisA vector for expression in S2 cells.

ELISA

Unmodified and acetylated H2A peptides diluted with Tris-buffered saline and Tween (TBST) buffer were immobilized on polystyrene plates for 2 hours at RT and then blocked with 5% BSA in TBST for 16 hours at 4 °C. After washing three times with TBST, purified antibodies in TBST were added and incubated for 1 hour at RT. After three washings in TBST, horse radish peroxidase (HRP)-conjugated anti-rabbit IgG was added and incubated for 1 hour at RT. After a further four washings, 2.5 mg/ml orthophenylenediamine, 0.002% H_2O_2 in 24 mM citric acid, and 130 mM Na_2HPO_4, pH 5.0, were added, and the reaction was stopped by adding 1 M H_2SO_4.

Supplementary Figure Legends

Supplementary Figure 1

(a) Western blotting showing the specificity of the anti-SMARCAD1 and anti-CBP antibodies. Whole-cell lysate of S2 cells (lanes 1, 3) and embryos (lane 2) were analyzed by SDS-PAGE and western blotting, performed using anti-SMARCAD1 (left

panel) or anti-CBP antibodies (right panel). (b) ELISA analysis of acetylated H2A antibodies using unmodified peptide, K5-acetylated H2A 1–9 peptide, or K8-acetylated H2A 4–12 peptide. Unmodified and acetylated peptides were immobilized on 96-well plates, and the ELISA was performed using purified anti-H2A AcK5 and anti-H2A AcK8 antibodies (bottom). (c) The level of histone acetylation was analyzed by western blotting with purified anti-H2A AcK5 and anti-H2A AcK8 antibodies using whole lysates from different embryonic developmental stages. (d) Sequence alignment of the N-termini of *Drosophila* canonical H2A and *Drosophila* H4. (e) *In vitro*-acetylated native core histones were blotted onto PVDF membranes and subjected to N-terminal automated sequencing.

Supplementary Figure 2

Edman degradation of the N-terminus of recombinant dH2A (a) and that of recombinant dH2B (b) yielded the sequence SGRGKGGKVK and the mixture of MPPKTSGKAA and PPKTSGKAAK sequences, respectively. The chromatogram of the PTH standards and the products of the first ten Edman degradations are shown.

Supplementary Figure 3

Confirmation of knockdown efficiency by RT-qPCR and western blotting. (a) RT-qPCR analysis of SMARCAD1 (left panel) and CBP (right panel) mRNA extracted from S2 cells treated with EGFP (as a control), SMARCAD1, and CBP dsRNAs. Values were normalized to dACT79B as a control. (b) Western blotting of whole-cell extracts of S2 cells after 3-day treatment with EGFP, SMARCAD1, and CBP dsRNAs was performed (left). Whole-cell extracts of S2 cells after 3-day treatment with EGFP, SMARCAD1, and S2 cells stably expressing EGFP–SMARCAD1 were analyzed by western blotting (right). Amido black staining of the membrane was used as a loading

control. (c) ChIP-qPCR analysis of normal rabbit IgG enrichment at the indicated promoter. qPCR following ChIP was performed using TSS-specific primers. (d) RT-PCR analyses of *dAct79B*, *SMARCAD1*, and *CG12477* levels in S2 cells transfected with EGFP, EGFP–SMARCAD1, EGFP–H2Awt, or the EGFP–H2A(K5A, K8A) mutant.

Supplementary Figure 4

Distribution of SMARCAD1 binding sites. (a) The genome-wide occupancy of SMARCAD1 (left panel) and CBP (right panel) and the percentage of the total number of sites (bottom table). The genome was partitioned into six discrete regions, based on annotated UCSC coordinates: downstream, downstream of the TES; includeFeature, containing the gene-coding sequence; inside, intergenic; overlapEnd, overlapping with the TES; overlapStart, overlapping with the TSS; and upstream, upstream of the TSS. (b) Functional categories of genes bound by SMARCAD1 showing the P value for the molecular function (upper table) as well as the biological process GO terms (bottom panel). (c) The SMARCAD1- and CBP-concentrated regions were determined using ChIPpeakAnno. (d) Target genes of SMARCAD1 and CBP in S2 cells: genome browser views. ChIP-seq signals are shown for anti-SMARCAD1 and anti-CBP antibodies after loading BedGraph (bdg) files on the Integrated Genome Browser.

Supplementary Tables

Supplementary Table 1

List of peptide sequences corresponding to Fig. 1d. Sequences further verified by LC–MS/MS are indicated.

Supplementary Table 2

List of primer sequences using RT-qPCR and ChIP-qPCR.

Supplementary Table 3

Microarray dataset from S2 cells transfected with control vector or SMARCAD1.

Supplementary Table 4

Microarray dataset from S2 cells treated with dsRNA from the control, SMARCAD1, or CBP.
